# An easy, reproducible and cost-effective method for andrologists to improve the laboratory diagnosis of non-obstructive azoospermia: a novel microcentrifugation technique

**DOI:** 10.1590/S1677-5538.IBJU.2015.0090

**Published:** 2016

**Authors:** Rosa Alice Casemiro Monteiro, Juliana Risso Pariz, Patrícia de Campos Pieri, Jorge Hallak

**Affiliations:** 1 Androscience – Pesquisa Clínica de alta Complexidade e Laboratório de Andrologia, São Paulo, Brasil;; 2 Seção de Andrologia - Divisão de Urologia, Faculdade de Medicina da Universidade de São Paulo, São Paulo, Brasil;; 3 Unidade de Reprodução Toxicologia - Departamento de Patologia, Faculdade de Medicina da Universidade de São Paulo, São Paulo, Brasil

**Keywords:** Non-obstructive, Azoospermia, Spermatozoa, Semen, Infertility, Male

## Abstract

This study describes a new method of microcentrifugation as an improved, viable, cost-effective option to the classical Cytospin apparatus to confirm azoospermia. Azoospermic semen samples were evaluated for cryptozoospermia by a centrifugation method similar to that of World Health Organization guidelines (2010; entire specimen centrifuged at 3000g for 15 min, and aliquots of the pellet examined). Then, if no sperm were detected, the pellet from that procedure was resuspended in culture medium, centrifuged (2000g for 15 min), and the entire pellet spread on a 4 X 6mm area of a slide and stained using the Christmas tree method (Nuclear-Fast solution and picric acid). The entire stained area was examined for the presence or absence of sperm. A total of 148 azoospermic samples (after standard WHO diagnosis) were included in the study and 21 samples (14.2%) were identified as sperm-positive. In all microcentrifugation slides, intact spermatozoa could be easily visualized against a clear background, with no cellular debris. This novel microcentrifugation technique is clearly a simple and effective method, with lower cost, increasing both sensitivity and specificity in confirming the absence or presence of spermatozoa in the ejaculate. It may represent a step forward of prognostic value to be introduced by andrology laboratories in the routine evaluation of patients with azoospermia in the initial semen analysis.

## INTRODUCTION

Non-obstructive azoospermia (NOA) affects approximately 10% of all men presenting with infertility and is responsible for 80% in the subgroup of azoospermic men, whereas obstructive azoospermia contributes to 20% of this subpopulation of infertile men (1). The management of patients with NOA relies on the correct diagnosis, induction of spermatogenesis to try to produce an ejaculate of viable spermatozoa, and finally techniques for sperm procurement, such as micro-dissection testicular sperm extraction, simple sperm extraction (TESE) or Fine Needle Aspiration (FNA), preferentially followed by either sperm or testicular tissue cryopreservation to subsequent intracytoplasmic sperm injection (ICSI) use (2-5).

‘‘Should only a few or no spermatozoa be seen at initial evaluation, the sample must be centrifuged and the sediment examined for spermatozoa. The term azoospermia can only be used if no spermatozoa have been found in the sediment’’ wrote Eliasson 1981 (3). Although apparently simple, the diagnosis of azoospermia has a wide variety of confounding factors including those related to methodology and different evaluation protocols, large errors associated with counting few spermatozoa, the number of microscopy fields to be analyzed, difficulties in examining debris-laden pellets (6). The World Health Organization`s recommended changes include, examining fixed uncentrifuged samples and indicating the sensitivity of the counting methods employed; however, existing centrifugation methods necessary for accumulating sufficient number of cells are also included (6).

Whereas that presence of any spermatozoa in a complementary test of azoospermic patient may determine the clinical approach to be adopted, the centrifugation method of cell suspension on slides (Shandon CytoSpin III Cytocentrifuge, Thermo Scientific, Waltham, MA, USA) is widely used to concentrate the ejaculate in a single droplet to enable checking for the presence of spermatozoa in an optical microscope (7, 8). The Cytospin apparatus is a bench-top centrifuge with a specially-designed rotor, and sample chambers in which a special micro-slide is vertically placed after being filled with 0.1mL of well-mixed whole semen and an equal volume of sterile saline added in situ. This method of diluting the specimen is preferable to using a premixed dilution and minimizes cell-sampling errors, and should be used on specimens in which “no spermatozoa” were detected in a wet preparation. After centrifugation the cells are deposited in a uniform monolayer in a compact area (32mm^2^) and even with the use of stains such as nuclear fast red and picroindigocarmine (described as NF-PICS or Christmas Tree stain; Sigma-Aldrich, St. Louis MO, USA) (9, 10), is not always easy to identify isolated sperm heads owing to the large amount of cellular debris. Therefore, our group has developed a simple and cost-effective technique as an alternative to the Cytospin method: the microcentrifugation technique to confirm azoospermia (Labnet, Woodbridge NJ, USA). The aim of this study is to demonstrate an alternative method to confirm and improve the laboratory diagnosis of non-obstructive azoospermia.

## PATIENTS AND METHODS

### Study population

A retrospective study was performed involving 148 slides from semen of non-obstructive azoospermic patients in the reproductive age (mean 40.66; standard deviation 9.40) presenting to male infertility evaluation at Androscience, High Complexity Clinical and Research Andrology Laboratories, São Paulo, Brazil, between November 2008 and July 2013. Institutional Review Board approval was obtained from the University of São Paulo Research Ethics Committee and before that all samples were collected after informed consent signature.

### Seminal Analysis

Semen sample was obtained by masturbation after 48 to 72 hours of sexual abstinence. All semen analysis was performed manually by the same investigator (RACM).

After liquefaction, macroscopic and microscopic parameters were analyzed according to World Health Organization (WHO) guidelines of 1999 and 2010 (6, 11). Semen was evaluated for cryptozoospermia by a centrifugation method similar to that in WHO (2010; entire specimen centrifuged at 3000g for 15 min, and aliquots of the pellet examined), following by triplicate sediment evaluation (100µL) in all field of Neubauer chamber (6). If any spermatozoa were present in the pellet obtained after this first centrifugation the sample was classified as cryptozoospermic and excluded. In the absence of spermatozoa after the first centrifugation, a standard WHO diagnosis of azoospermia was given and the samples were further alternately processed by the microcentrifugation technique, proposed by this study.

The samples had all of the sediment left after the first centrifugation resuspended in a small amount (100µL) of Human Tubal Fluid (Modified HTF Medium, Irvine Scientific, Santa Ana, CA, USA) and centrifuged in a mini-centrifuge (Labnet, Woodbridge NJ, USA) at 2000g for 15 minutes. After removal of the supernatant, the pellet was deposited on and gently spread over a glass slide with a pipette in order to cover an area of 2 to 4cm^2^.

The slides obtained were then fixed in absolute ethanol for 15 minutes, and air dried. Nuclear-Fast Red solution [73.1mM Aluminum Sulfate (cat#202614, Sigma Aldrich, St. Louis, MO, USA) and 1.49mM Fast Nuclear Red (cat#N8002, Sigma Aldrich, St. Louis, MO, USA) in 50mL distilled water], was placed over the slide for 15 minutes, after which the slides were carefully rinsed with distilled water, covered with picroindigocarmine stain [7.15mM Indigo Carmine (cat#18130, Sigma Aldrich, St. Louis, MO, USA) and 50mL of Picric Acid (cat#92540, Sigma Aldrich, St. Louis, MO, USA)] for 15 seconds, immediately rinsed with absolute ethanol, left to air dry, covered with coverslips and mounted with Entellan (Merck Millipore, Darmstadt, Germany) (8).

The slides were examined at 1000×magnification in optical microscope (Nikon Eclipse E200, Japan) for the following parameters: sperm presence or absence, sperm integrity, and for the presence of cellular debris.

### Statistical analysis

Statistical analysis was performed using IBM SPSS Statistics 19 for Windows. We calculated the mean of age (years) and frequency of events in the study (%).

## RESULTS

**Figure 1:**
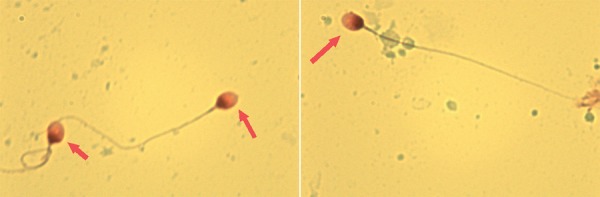
Spermatozoa found after microcentrifugation technique (2000g for 15 minutes), stained by nuclear fast red and picroindigocarmine (described as NF-PICS or Christmas Tree stain) in an azoospermic man. We observed clear slides obtained with sperm integrity preserved, without cellular debris.

A total of 148 azoospermic samples (after standard WHO diagnosis) were included in the study. Twenty one samples (14.2%) were identified as sperm-positive. In addition, intact spermatozoa could be visualized against a clear background, with minimal cellular debris in all slides (Figure-1).

1000x in immersion oil.

## DISCUSSION

Although intracytoplasmic sperm injection (ICSI) is a remarkably effective technique in allowing fatherhood in men previously considered sterile, sperm retrieval from the epididymis or testis should be the last option to be given to men. Gnoth et al. showed that the source of spermatozoa has not relevant impact on the results of ICSI cycles as long as fresh motile, morphologically normal spermatozoa are used, proposing, in case of cryptozoospermia, to preferentially use ejaculated spermatozoa to prevent those men from an unnecessary testicular biopsy avoiding risks and costs implied (12). In addition, the use of a single sperm found in ejaculate in ICSI resulting in successful pregnancy was demonstrated by Desai et al. (13). Thus, azoospermia requires a diligent search for reversible factors and treatment to restore natural fertility and a pragmatic ‘default to ICSI’ can be avoided (14).

Semen analysis is the single most important diagnostic tool method for the assessment of male infertility and the gold standard according to the World Health Organization. The WHO guidelines offer detailed advice on every aspect of semen analysis and consequently has become the gold standard for the field (6). According to these guidelines, a patient is diagnosed as azoospermic when few or no sperm cells are found in wet preparations, the sample is centrifuged and there is also absence of sperm in the sediment (3). However, the presence or not of spermatozoa in the sediment depends on the centrifugation time and speed and on how much of the sediment is examined and how thoroughly (6). No technique more stringent is proposed by the WHO guidelines to characterize the sample further after the routine seminal analysis does not identify any spermatozoa.

Shandon Cytospin emerged in the 1970s as an apparatus suited for cell concentration and has since been used for sediment analysis of many fluids. In the late 1980s it was first used to analyze the presence of germ cells exfoliate in semen samples and identification of carcinoma in situ of the testis (15). Afterwards Cystospin was introduced in Andrology laboratories as an additional test to confirm azoospermia. Despite being a simple and rapid method, processing the sample often causes damage to the structure of the sperm cells, such as the separation of the heads from mid piece and tail. Furthermore, the presence of cellular debris makes a detailed analysis difficult and time-consuming. In the view of these disadvantages, the microcentrifugation technique developed by our group proved to be as effective and possibly increased the sensitivity and specificity in confirming azoospermia, beyond the cost 5 times lower, since it uses the whole sediment obtained after the first centrifugation, minimizing cell losses but maximizing debris washout.

We purposed to demonstrate a method detection of single sperm cells in semen sediments as Cytospin, but with visualization and identification of isolated sperm cells much easier. From the technical standpoint, the differences between the two methods presented here are: a second wash to remove cellular debris, the rotational speed applied to the sample, and the way the final sample is “deposited” on the slide. More important than the time and rotation speed, the new technique proposed uses another wash with culture medium which can contribute to both the concentration of the sediment and the removal of cellular debris. The centrifugation and pellet resuspension technique is widely used in laboratory routine to eliminate cell debris and was applied in our method. Additionally, the sample deposition on slide is carried out differently: while Cytospin method concentrated all sample in a small area of slide, the microcentrifugation allows the sample to spread over a larger area of slide surface with a pipette.

There is no consensus on which rotation is best for the processing of azoospermic semen samples. Whereas the Cytospin centrifuge uses 700g for five minutes, the microcentrifugation proposed by us used 2000g for 15 minutes. In the literature, the speed centrifugation of semen samples remains controversial. Corea and colleagues (16) showed that centrifugation should apply at least 1000g for 5 minutes in order to find sperm cells in suspected cases of azoospermia but the WHO guidelines (6) suggests centrifugation at 3000g for 15 minutes for samples in which no sperm cell is found. Other studies showed that centrifugation at 1000g for 15 minutes is effective (17, 18). Our hypothesis of 2000g for 15 minutes was based on laboratory practice and literature, considering that (I) little centrifugation time was not enough for the pellet formation and (II) 3000g damaged the sperm integrity.

The NF-PICS or Christmas Tree stain is one of the most widely used for histological tests for the identification of sperm in sexual assault cases (10). With this stain it is possible to color both the post-acrosomal region of the sperm head, that stains pink, and the acrosome itself, that stains light pink, while cell debris are colored green, allowing easy differentiation of spermatozoa from cellular debris and thus facilitating the reading of the slide. Other cellular dyes, such as eosin-nigrosin, Hoechst 33342 fluorescent stain and Diff-Quick can be suggested to replace the NF-PICS (6, 19). Therefore, as azoospermia is a complex diagnosis and subjected to doubts, it is important to preserve the slide with mounted coverslip after staining not only for subsequent reading but also to make it available for a second opinion review by other professionals.

In conclusion, the microcentrifugation method developed by our group showed to be a simple, effective, and low cost technique able to increase the sensitivity of confirming azoospermia, an important step of prognostic value to be used hand in hand with clinical and surgical approaches aimed to reverse azoospermic state.
