# Supplementary material for: An easy, reproducible and cost-effective method for andrologists to improve the laboratory diagnosis of non-obstructive azoospermia: a novel microcentrifugation technique
Source: Int Braz J Urol. 2016 Jan-Feb;42(1):132–8. doi: 10.1590/S1677-5538.IBJU.2015.0090 (PMC4811238; doi:10.1590/S1677-5538.IBJU.2015.0090)
Supplement: Related texts

# Reply: EDITORIAL COMMENT

10.1590/S1677-5538.IBJU.2015.0090.1

In this issue of Int Braz J Urol, a paper by Monteiro and colleagues reports on a novel semen analysis method to increase precision in the diagnosis of azoospermia (1). This paper is important given the recent changes in the World Health Organization (WHO) laboratory methods for evaluation of human semen (2) and the current discussion on how to count sperm properly (3). This editorial commentary is aimed to highlight Monteiro and colleagues findings and to expand the discussion on the clinical importance of laboratory andrology.

Azoospermia is a laboratory diagnosis of ejaculates that lack spermatozoa (4). Despite being a descriptive term that does not imply any specific underlying cause, the absence of spermatozoa in a semen analysis has many ramifications for both clinical practice and research. The reliable diagnosis of azoospermia is important not only for male infertility and to ensure the success of vasectomy but also for assessing efficacy of male contraceptive trials. Moreover, the confirmation for the presence of semen in forensic studies is based on the direct identification of spermatozoa.

Seminal fluid is a complex mixture of secretions from the seminal vesicle, the prostate gland and the combined contribution of the epididymis, testicles and bulbourethral glands (5). An average male ejaculates around 3.2 mL of semen. Each milliliter contains approximately 64 million spermatozoa (6). This number can vary with the age of the male, and may be affected by other factors, including medical conditions, genetic background, ejaculatory abstinence and life-style habits such as diet, tobacco and illicit drug use (7). On the contrary, azoospermia affects approximately 1% of males in the general population and 10-15% of those facing infertility (8).

In the context of infertility, men with azoospermia do not have an unattainable potential to initiate a pregnancy. From the laboratory standpoint, the assessment of an initially azoospermic ejaculate should be followed by the examination of the pelleted semen to exclude cryptozoospermia, which is defined by the presence of very small number of live sperm (8). This is important because the finding of live sperm may allow intracytoplasmic sperm injection to take place without the need of surgical sperm retrieval. Since the diagnosis of azoospermia or cryptozoospermia is based upon semen analysis, proper laboratory techniques are crucial to reduce analytical errors and enhance precision (9).

Accuracy, the degree to which the measurement reflects the true value, and precision, the reproducibility of the results, are important for clinicians who rely upon the values provided by the laboratory to direct the further work-up and to counsel men seeking fertility (9). Interestingly, data from surveys of laboratory practice in the United States and the United Kingdom revealed that semen analysis techniques are poorly standardized. Interestingly, less than half of the laboratories providing semen analysis services have implemented quality control practices and only about 5% fully complied with the WHO manual for the laboratory assessment of human semen (10, 11).

Owed to its complex nature, we have advocated that semen analysis should be ideally carried out in Andrology Laboratories equipped with experienced technicians and validated systems, and enrolled in internal and external quality control programs (3, 8, 9, 12). In this current issue of Int Braz J Urol, Monteiro and colleagues shed light on this very same issue (1). The authors studied semen specimens from men with nonobstructive azoospermia, all of which were negative for the presence of sperm after processing according to the new WHO guidelines. Of note, the WHO method recommends centrifugation of a 1 mL aliquot of semen for 15 minutes using high *g* force and examination of two aliquots of the pelleted semen (2). The use of high centrifugation force is warranted because the accuracy of any centrifugation protocol using low g forces in pelleting all spermatozoa in a given ejaculate is uncertain (13). However, the aforementioned WHO method is flawed by not recommending examination of the whole specimen. Furthermore, the pelleted semen gives rise to large amount of debris that might make it difficult to identify isolated spermatozoa (13). Monteiro et al. proposed an additional centrifugation step using high g force, followed by examination of the whole pellet deposited in a large area of a microscope glass slide (1). For this, the slides were fixed and stained with nuclear fast red and picroindigocarmine. Interestingly, clear slides were obtained with minimum debris, and isolated spermatozoa were identified in about 14% of the specimens evaluated. These patients were reclassified as having cryptozoospermia, with obvious diagnostic and management implications.

Although a comparative analysis with other centrifugation and staining techniques has not been included, Monteiro and colleagues’ method was clearly superior to the WHO method for evaluating azoospermic specimens. The finding of any sperm in a semen analysis meticulously performed, such as the one object of this commentary, has important implications for men with nonobstructive azoospermia. It is not only a proof that residual sperm production exists, thus allowing the affected men to achieve biological offspring with the aid of assisted reproductive technology, but also imply that sperm retrieval is likely to be successful whenever required.

Collectively, the study of Monteiro and colleagues highlights the importance of semen analysis in the context of azoospermia and the role of Andrology Laboratories to improve precision and reproducibility of reported results.

*Sandro C. Esteves, MD, PhD* *Medical and Scientific Director, ANDROFERT* *Center for Male Reproduction* *Av. Dr. Heitor Penteado 1464* *Campinas, SP, 13075-460, Brazil* *E-mail: s.esteves@androfert.com.br*
